# Supplementary material for: Structure of the malaria vaccine candidate antigen CyRPA and its complex with a parasite invasion inhibitory antibody
Source: eLife. 2017 Feb 14;6:e20383. doi: 10.7554/eLife.20383 (PMC5349852; doi:10.7554/eLife.20383)
Supplement: Supplementary file 1. — DOI: http://dx.doi.org/10.7554/eLife.20383.017 [file elife-20383-supp1.docx]

**Supplementary file 1. Data collection and refinement statistics**

|  | c12-hexagonal (5ezi) | c12-orthorhombic (5ezj) | c12-monoclinic (5ezl) | PfCyRPA (5ezn) | PfCyRPA/c12 (5ezo) |
| --- | --- | --- | --- | --- | --- |
| **Data collection** |  |  |  |  |  |
| Space group | P6_5_22 | P2_1_2_1_2_1_ | C2 | P2_1_ | P2_1_ |
| Cell dimensions |  |  |  |  |  |
| *a*, *b*, *c* (Å) | 82.7, 82.7, 361.9 | 64.8, 72.8, 109.9 | 101.4, 108.9, 97.4 | 46.2, 78.5, 97.0 | 95.8, 44.7, 121.3 |
| α, β, γ (°) | 90.0, 90.0, 120.0 | 90.0, 90.0, 90.0 | 90.0, 97.8, 90.0 | 90.0, 94.9, 90.0 | 90.0, 108.0, 90.0 |
| Resolution (Å) | 50.9 - 1.61  (1.67 - 1.61)* | 41.9 - 1.95  (2.02 - 1.95) | 48.3 - 2.43  (2.52 - 2.43) | 43.3 - 2.5  (2.59 - 2.50) | 44.7 – 4.0  (4.2 – 4.0) |
| *R*_meas_ (%) | 10.9 | 12.8 | 15.1 | 10.5 | 42.0 |
| *I* / σ*I* | 8.5 (0.8) | 10.5 (1.0) | 7.7 (1.0) | 12.4 (0.7) | 3.3 (1.2) |
| Completeness (%) | 100 (100) | 98 (100) | 99 (100) | 100 (100) | 99.3 (99.3) |
| Redundancy | 13.8 (16.3) | 6.3 (6.7) | 3.4 (3.5) | 7.1 (7.2) | 3.3 (3.4) |
|  |  |  |  |  |  |
| **Refinement** |  |  |  |  |  |
| Resolution (Å) | 50.9 - 1.61  (1.67 - 1.61) | 41.9 - 1.95  (2.02 - 1.95) | 48.3 - 2.43  (2.52 - 2.43) | 43.3 - 2.50  (2.59 - 2.50) | 44.7 – 4.0  (4.6 – 4.0) |
| No. unique  reflections | 96028 (9389) | 38602 (3816) | 39276 (3924) | 24072 (2402) | 8602 (2678) |
| *R*_work_ / *R*_free_ | 19.4 / 22.1 | 19.8 / 24.7 | 18.7 / 24.5 | 18.3 / 25.0 | 24.9 / 31.2 |
| No. atoms |  |  |  |  |  |
| Protein | 3329 | 3320 | 6644 | 5313 | 5995 |
| Ligand/ion | 110 sugars / 1 Cl^-^ | 49 sugars | 42 sugars | 0 | 38 sugars |
| Water | 439 | 255 | 190 | 6 | 0 |
| *B*-factors |  |  |  |  |  |
| Protein | 40.3 | 48.8 | 47.0 | 103 | 76.2 |
| Ligand/ion | 65.9 / 39.3 | 92.1 | 80.3 | - | 112.1 |
| Water | 47.6 | 48.0 | 43.4 | 73.6 | - |
| R.m.s. deviations |  |  |  |  |  |
| Bond lengths (Å) | 0.006 | 0.007 | 0.010 | 0.009 | 0.004 |
| Bond angles (°) | 0.9 | 0.9 | 1.4 | 1.0 | 0.7 |

A single crystal was used for each structure.

*Values in parentheses are for highest-resolution shell.
