# Supplementary material for: Structure of the malaria vaccine candidate antigen CyRPA and its complex with a parasite invasion inhibitory antibody
Source: eLife. 2017 Feb 14;6:e20383. doi: 10.7554/eLife.20383 (PMC5349852; doi:10.7554/eLife.20383)
Supplement: Supplementary file 2. — Predicted interactions between PfCyRPA and the CDRs of mAb c12. The amino acid residues involved and the nature of the interactions are listed. DOI: http://dx.doi.org/10.7554/eLife.20383.018 [file elife-20383-supp2.docx]

**Supplementary file 2.** **Interactions between PfCyRPA and the c12 Fab**

| van der Waals interactions | | | | |
| --- | --- | --- | --- | --- |
| PfCyRPA residue | c12 residue  (CDR; # of contacts) | | | |
| Asn45 | Arg50 (CDR1_L_; 1) Asn51 (CDR1_L_; 3) Ser87 (framework; 5) | | | |
| Val46 | Arg50 (CDR1_L_; 4) | | | |
| Pro47 | Arg50 (CDR1_L_; 7) | | | |
| His76 | Tyr112 (CDR3_L_; 6) | | | |
| Tyr78 | Phe70 (CDR2_L_; 3) Asp52 (CDR1_L_; 4) | | | |
| Thr89 | Phe70 (CDR2_L_; 9) | | | |
| Leu90 | Phe70 (CDR2_L_; 2) | | | |
| Asn91 | Tyr120 (CDR3_H_; 6) | | | |
| Lys95 | Trp51 (CDR1_H_; 5) Tyr120 (CDR3_H_; 3) Tyr122 (CDR3_H_; 9) | | | |
| Glu96 | Tyr120 (CDR3_H_; 5) | | | |
| Phe136 | Tyr120 (CDR3_H_; 1) | | | |
| Tyr144 | Tyr120 (CDR3_H_; 4) | | | |
| Asn145 | Tyr120 (CDR3_H_; 2) | | | |
| Asn146 | Tyr120 (CDR3_H_; 3) | | | |
| Possible hydrogen-bonds | | |  | |
|  | **Light chain** |  | |  |
| Asn45 Nδ2 | Asn51 Oδ1 | |  | |
| Asn45 Oδ1 | Ser87 Oγ | |  | |
| Asp66 Oδ2 | Arg50 Nε | |  | |
| Glu74 Oε1 | Thr113 Oγ1 | |  | |
| His76 Nε2 | Tyr112 O | |  | |
| Tyr78 OH | Asp52 Oδ2 | |  | |
| Thr89 O | Asn73 Nδ2 | |  | |
|  | **Heavy chain** | |  | |
| Asp92 N | Tyr120 O | |  | |
| Tyr144 O | Tyr120 OH | |  | |

* CDR1_L_ denotes loop CDR1 of the light chain etc.
